# Prognostic role of vascular endothelial growth factor in cervical cancer: a meta-analysis

**DOI:** 10.18632/oncotarget.15044

**Published:** 2017-02-03

**Authors:** Jing Zhang, Jiaming Liu, Chenjing Zhu, Jialing He, Jinna Chen, Yunliu Liang, Feng Yang, Xin Wu, Xuelei Ma

**Affiliations:** ^1^ State Key Laboratory of Biotherapy and Cancer Center, West China Hospital, Sichuan University, and Collaborative Innovation Center for Biotherapy, Chengdu 610041, PR China; ^2^ Department of Neurosurgery, West China Hospital, Sichuan University, Chengdu 610041, PR China; ^3^ Department of Urology, Institute of Urology, Laboratory of Reconstructive Urology, West China Hospital, Sichuan University, Chengdu 610041, PR China

**Keywords:** VEGF, cervical cancer, prognosis

## Abstract

The prognostic role of vascular endothelial growth factor (VEGF) in cervical cancer is controversial to date. The aim of this study was to evaluate the prognostic value of VEGF and VEGF-C in patients with cervical cancer. Relevant studies were identified by systematic search of the PubMed and Embase database. The primary data of eligible studies was hazard ratio (HR) with 95% confidence interval (95% CI) of survival outcomes, including overall survival (OS), disease-free survival (DFS) and progression-free survival (PFS). Pooled HR (95% CI) was calculated to evaluate the prognostic role of VEGF and VEGF-C in cervical cancer patients. The methodological qualities of the included studies were assessed using REMARK. Fourteen eligible articles including 1306 patients were included in the meta-analysis. The pooled HRs (95% CIs) of VEGF for OS and DFS/PFS were 2.29 [1.27, 4.14] and 2.77 [1.37, 5.62], respectively. The HR (95% CI) of VEGF-C for OS was 3.94 [2.22, 6.99]. This meta-analysis suggested that high expressions of VEGF and VEGF-C were significantly associated with poor survival outcome in cervical cancer patients.

## INTRODUCTION

Cervical cancer is the third most common cancer and the fourth leading cause of cancer-related deaths in females worldwide. Globally, 529,800 new cancer cases were diagnosed and 275,100 cases died from the disease in 2008 [1]. Although cervical cancer incidence rates have declined significantly in developed countries recently, it remains to be one of the most common cancers in women in developing countries [1]. In order to improve survival outcome of cervical cancer, several prognostic biomarkers have been identified. Human-papilloma virus (HPV) related biomarkers have been proven as effective biomarkers in the diagnosis and prevention of cervical cancer [2, 3]. However, the researchers are still screening new markers which are highly associated with cervical cancer progression and prognosis.

Angiogenesis is considered as a very important biological process for primary cancer growth and metastasis. Vascular endothelial growth factor (VEGF) is a significant biomarker causing tumor angiogenesis. It has been demonstrated that the over expression of VEGF is associated with poor survival in various cancer patients, including lung cancer, colorectal cancer, ovarian cancer and some other tumors [4–7]. Meanwhile, several studies have evaluated the prognostic value of VEGF in cervical cancer. However, the results are conflicting or inconclusive. This discrepancy is mostly due to the relatively small sample size, different detecting methods and genuine heterogeneity. In this study, we sought to conduct a meta-analysis to evaluate the prognostic value of high expression of VEGF in patients with cervical cancer.

## RESULTS

### Eligible studies

**Figure 1:**
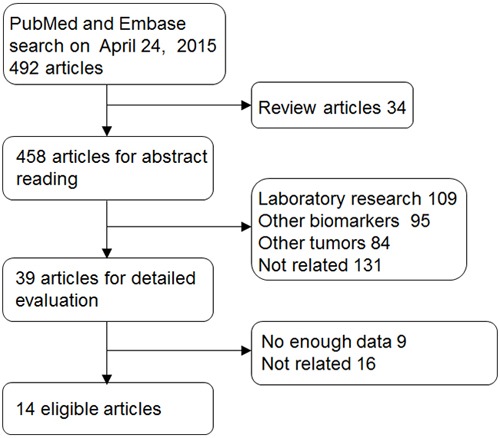
Selection of studies

The initial search yielded 492 articles from PubMed and Embase databases after duplicates were removed. The selection process was shown in Figure 1. Handy searches were also conducted for the reference lists of primary studies, review articles, and clinical guidelines. However, no additional eligible studies were retrieved. After screening the titles and abstracts, 453 studies were excluded. Then 25 articles were further excluded after full texts reading according to the criteria. Finally, 14 eligible articles [8–21] including 1306 patients containing survival outcomes were included.

### Baseline characteristics of studies included in the meta-analysis

These eligible studies were published from 2000 to 2011. The clinical characteristics of patients and other useful information have been listed in Table 1.

**Table 1:** Characteristics of all identified studies

author	year	N	median age	FIGO (I.II/ III.IV)	LNM (yes/no)	initial therapy	histological type	VEGF type	sampling site	sampling time	method	dilution	attitude	survival outcome	cut-off	quality
Cheng WF	2000	135	50	135/0	28/107	OP	SCC,Adeno-Ca,Ade, SCNEC	VEGF	tissue	post	IHC	NR	pos	OS,DFS	112 pg/ml	18
Gaffney DK	2003	55	NR	34/21	NR	RT	SCC,Adeno-Ca,Ade	VEGF	tissue	post	IHC	1:20	pos	OS,DFS	NR	12
Kang JO	2004	42	62	36/6	20/22	RT	SCC	VEGF	tissue	post	IHC	1:10	pos	OS	10%	13
Kim YH	2010	199	49	199/0	31/168	OP/OP+RT /OP+CCRT	SCC,Adeno-Ca,Ade	VEGF	tissue	NR	IHC	NR	NR	OS,DFS	NR	13
Lee IJ	2002	117	NR	117/0	25/92	OP/OP+RT/OP +CT/OP+RT+CT	SCC,Adeno-Ca,Ade	VEGF	tissue	post	IHC	1:50	pos	OS,DFS	50%	14
Loncaster JA	2000	100	49	71/29	NR	RT	SCC,Adeno-Ca,Ade	VEGF	tissue	post	IHC	1:200	pos	OS,PFS	NR	13
Randall LM	2009	173	39	173/0	146/27	RT/RT+CT	SCC,Adeno-Ca,Ade	VEGF	tissue	post	IHC	NR	neg	OS,PFS	NR	16
Choi CH	2008	46	50	29/17	11/18	NAC	SCC,Adeno-Ca	VEGF	tissue	post	IHC	1:100	pos	DFS	NR	12
Fujimoto J	2004	40	NR	40/0	NR	OP	SCC	VEGF-C	tissue	post	ELISA	1:50	pos	OS	300pg/ml	11
Ma DM	2011	82	50	82/0	36/46	OP+RT	SCC,Adeno-Ca	VEGF-C	tissue	post	RT-PCR	NR	pos	OS	NR	12
Ueda M	2002	52	50	52/0	41/11	OP+RT/OP+CT	SCC,Adeno-Ca,Ade	VEGF-C	tissue	post	IHC	NR	pos	OS	50%	12
Hashimoto I	2001	75	53	61/14	61/14	OP/OP+RT/OP+CT /RT/CCRT	SCC,Adeno-Ca,Ade	VEGF-C	tissue	pre	IHC	NR	pos	DFS	NR	14
		53	50	53/0	38/15	16/37	OP/OP+RT/OP+CT	SCC,Adeno-Ca			post					
Bachtiary B	2002	23	72	18/5	8/15	RT	SCC,Adeno-Ca	VEGF	serum	post	ELISA	NR	pos	PFS	224pg/ml	16
Zusterzeel PL	2009	167	42	151/16	23/102	OP/OP+RT/CCRT/RT/ palliative	SCC,Adeno-Ca,Ade	VEGF	serum	post	ELISA	NR	NR	OS,DFS	5pg/ml	16

N number of patients; NR, not reference; FIGO International Federation of Gynecology and Obstetrics staging; LNM lymph node metastasis; OP operation; RT radiation therapy; CCRT concurrent chemoradiation; CT chemotherapy; NAC neoadjuvant chemotherapy; SCC, squamous carcinoma; Adeno-Ca, adenosquamous carcinoma; Ade, adenocarcinoma; SCNEC, small cell neuroendocrine carcinomas; G1,2,G3, histological different grade; VEGF, vascular endothelial growth factor; IHC, immunohistochemistry; ELISA, enzyme-linked immunosorbent assay; RT-PCR, reverse transcription-polymerase chain reaction; pos, positive; neg, negative; OS, overall survival; DFS, disease free survival; PFS, progression free survival

### Survival outcomes

**Figure 2:**
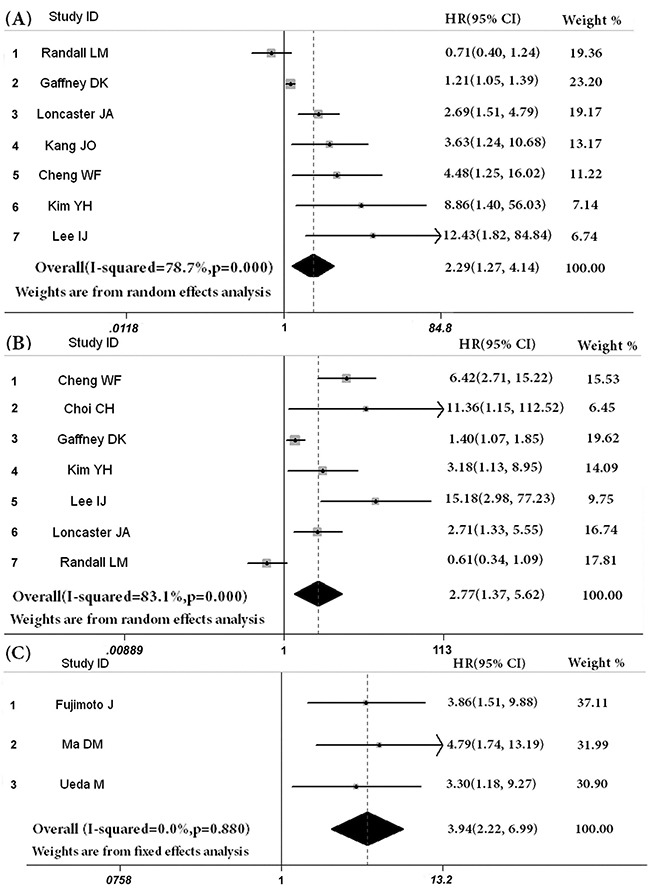
Estimated hazard ratios (HRs) and 95% CIs summary **A**. overall survival of VEGF expression in cervical cancer, **B**. disease free survival or progression free survival (DFS/PFS) of VEGF expression in cervical cancer, **C**. overall survival of VEGF-C expression in cervical cancer.

Seven studies [8, 12, 14–17, 19] including 821 patients were used to evaluate the relation of VEGF expression and overall survival. Figure 2 showed that the pooled HR (95% CI) of these studies for OS was 2.29 [1.27, 4.14] (heterogeneity: I^2^ = 79%, P<0.001). The pooled HR (95% CI) of 7 studies [8, 10, 12, 15–17, 19] for DFS/PFS was 2.77 [1.37, 5.62] (n=825, I^2^ = 83%, P<0.001). We also performed a meta-analysis on the prognostic role of VEGF-C in cervical cancer tissue. The combined HR (95% CI) of three studies [11, 18, 20] for OS was 3.94 [2.22, 6.99] (heterogeneity: I^2^ = 0%, P=0.88). Only one study reported the DFS of VEGF-C in cervical cancer [13], and the HR (95% CI) for DFS in this study was 1.93 [0.96, 3.92]. Two studies reported that high serum VEGF levels in cervical cancer could be used to predict poor survival outcome. The pooled HR (95% CI) of high serum VEGF for DFS/PFS was 2.67 [1.53, 4.64] [9, 11]. Meanwhile, one study reported that the OS was the important endpoint. The HR (95% CI) of serum VEGF [21] for OS was 1.92 [1.01, 3.64]. All above results of meta-analysis were reviewed in Table 2.

**Table 2:** HR (95% Cl) for VEGF and VEGF-C to predict the survival outcome

	Survival outcome	Study n.	Patient n.	Model	HR (95% Cl)	P value	Heterogeneity (I^2^,p)	Conclusion
VEGF	OS	7	821	Random	2.29 [1.27, 4.14]	0.006	79%, <0.0001	Positive
VEGF	DFS/PFS	7	825	Random	2.77 [1.37, 5.62]	0.005	83%, <0.00001	Positive
VEGF-C	OS	3	174	Fixed	3.94 [2.22, 6.99]	<0.00001	0,0.88	Positive
VEGF-C	DFS	1	128	——	1.93 [0.96, 3.92]	0.07	——	——
sVEGF	OS	1	167	——	1.92 [1.01, 3.64]	0.05	——	——
sVEGF	DFS	2	190	Fixed	2.67 [1.53, 4.64]	0.0005	34%,0.22	Positive

VEGF, vascular endothelial growth factor; sVEGF, serum VEGF; OS, overall survival; DFS, disease free survival; PFS, progression free survival; n, number.

### Potential publication bias

Begg's test and funnel plot was used to evaluate the publication bias. No significant publication bias was found in the meta-analysis of VEGF for OS or DFS/PFS. The p-values of Begg's test were 0.051 and 0.176, respectively. We also did the Begg's test in the other groups, and publication bias was not found either.

### Assessment of quality

Overall, the global qualitative score of the included studies ranged from 55.0% to 90.0% with a mean score of 68.57%. Concerning the global score, there was no statistically significant difference between the 12 positive [8–12, 14–18, 20, 21] and the 2 negative [13, 19] trials (mean score, 67.5% versus 75%, p =0.531). The difference was not significant in qualitative scores between these fourteen studies with positive and negative conclusion (Mann Whitney test, p=0.312).

## DISCUSSION

The prognostic values of VEGF expression have been proven in the recent meta-analyses in gastric cancer, breast cancer, lung cancer and many other cancers. As far as we know, this study was the first meta-analysis revealing the prognostic role of VEGF in cervical cancer. The combined results suggested thathigh expressions of VEGF and VEGF-C were significantly associated with poor survival outcome in patients with cervical cancer. In this meta-analysis, the pooled HRs (95% CI) of VEGF expression for OS and DFS/PFS were 2.29 [1.27, 4.14] and 2.77 [1.37, 5.62], respectively. The intervals of the HRs did not overlap 1, which showed that VEGF could predict the prognosis of cervical cancer patients. As a rule of the thumb, RR>2 suggests that the prognostic factor is useful in clinical practice [22]. The results suggested that high expression of VEGF could be considered as a useful predictive biomarker in cervical cancer. Besides, Sun et al. [23] recently performed a meta-analysis to investigate the association between VEGF expression and lymph node metastasis (LNM) in cervical cancer. They found that VEGF-positive expression was related with higher risk of LNM in cervical cancer, with the odds ratio of 2.87 (95% CI = 1.85-4.44, P < 0.001). Thus, our findings could be partly explained that worse survival might be associated with higher risk of LNM in cervical cancer patients with VEGF-positive expression. Furthermore, Food and Drug Administration (FDA) approved the use of bevacizumab in cervical cancer in 2014 [24] suppressing the biological activity of VEGF, which supports our conclusion from the indirect side.

Although there was significant heterogeneity (I^2^>50%) between studies in different groups, we have strictly grouped the eligible studies by location, detecting method, cut-off value and VEGF subtype. However, these entire attempts could not eliminate the heterogeneity. Considering these results of subgroup analysis, the heterogeneity of this meta-analysis might be due to the multiply influence of location, detecting method, cut-off value, VEGF subtype and other characteristics.

Three studies [11, 18, 20] were eligible for meta-analysis of prognostic value of VEGF-C in cervical cancer. The combined HR (95% CI) of VEGF-C was 3.94 [2.22, 6.99] in fixed effect model. No significant heterogeneity has been found (p=0.88, I^2^=0%). Nowadays, VEGF-C has been proven to induce selective hyperplasia of the lymphatic vasculature, which is involved in immune function, inflammation, and tumor metastasis.

This meta-analysis had some limitations. First and foremost, Tierney's method [25] has proved the method is not perfect to calculate the studies based on the Kaplan-Meier survival curve and p-value. We deliberated the data of every article intensively in order to find unreasonable results to rule out. Fortunately, we did not find unreasonable results in these articles. Secondly, we used the software designed by Matthew Sydes and Jayne Tierney [25] to calculate the logHR and SE, which retained only percentile. At the same time, we verified the data again by using Revman, and only minimal bias was observed. Thirdly, although we tried to optimize standardization, some remaining variability like cut-off value in definitions was unavoidable. The cut-off value of VEGF expression could not be in an agreement. Due to lacking of abundant VEGF expression data in global population, it is difficult to set a standard cut-off value.

The publication bias is a major problem in the meta-analysis. Begg's test was chosen in our investigation. We did not find evidence that publication bias might significantly influence our results. However, it should be noted that our meta-analysis could not completely exclude biases. For example, positive results tend to be accepted by journals, while negative results are often rejected or even not submitted, which probably introduced bias.

In conclusion, over expression of VEGF or VEGF-C was associated with poor survival in patients with cervical cancer. These findings suggested that VEGF inhibition therapy could have an important role in cervical cancer, and that VEGF might be routinely examined to predict prognosis in cervical cancer patients. However, more investigations are warranted to further verify our results.

## MATERIALS AND METHODS

### Search strategy

The electronic databases PubMed and Embase were searched for potential studies on April 24th, 2015. The following key words were used to retrieve titles and abstracts, [cervical] AND ([cancer OR tumor OR carcinoma]) AND ([VEGF] OR [vascular endothelial growth factor]).

### Study inclusion/exclusion criteria

Studies were considered eligible if they met all the following inclusion criteria, (i) patients had any type of cervical cancer, (ii) researchers measured the expression of VEGF in tissue or serum, and (iii) studies investigated available data involving the prognostic role of VEGF in cervical cancer patients with survival outcome (OS, DFS/PFS).

Studies were excluded if any following conditions existed, (i) review articles, non-human studies or letters, (ii) duplicated publications, (iii) or lack of adequate information to calculate log hazard ratio (logHR) and SE, following Parmar or Tierney's methods [25, 26]. Abstracts and full texts were reviewed for all searched papers, and reference list were searched for potentially eligible studies according to the above criteria. To avoid duplication data, if more than one study was completed in one particular center, only the biggest one was used.

### Data extraction

Two reviewers independently determined study eligibility by reviewing the abstracts and full texts. Disagreements were resolved by consensus. The primary data was survival ratio of VEGF in cervical cancer patients, including hazard ratio (HR) and 95% confidence interval (CI), or the Kaplan–Meier survival curves with log-rank p value. Additional data extracted from the studies included first author, publication year, study size, age, tumor stage, tumor size, lymph node metastasis status, initial therapy, histological classification, methods to detect VEGF, cut-off value, the attitude of conclusion and other clinical characteristics.

### Statistical methods

The logHR and SE (logHR) (SE) were used for aggregation of the survival results. However, these statistical variables were not available directly in some studies. We calculated the accessible statistics on the basis of available data with methods developed by Parmar [26] and Tierney [25] instead. The pooled outcomes were presented by Forest plots for estimation of the prognostic value of VEGF expression. Statistical heterogeneity was defined as significant if Q test with p<0.10 or I^2^≥50%. If there was no significantly statistical heterogeneity (p≥0.10 and I^2^<50%), a fixed effect model was used for meta-analysis. Otherwise, the random effect models were used in the study [27]. A pooled HR>1 indicated worse outcome for the high expression of VEGF, meanwhile, it would be considered statistically significant if the 95% CI did not overlap 1. The Begg's tests and funnel plots were applied to assess the potential publication bias. If p>0.05, it was considered that there was no significant publication bias [28]. All above calculations were evaluated by using STATA 11.0 (STATA Corporation, College Station, TX).

### Assessment of quality

An assessment of study methodology was made according to the previously defined criteria [29]. These principles were used to define 20 individual study characteristics, which were deemed to be the key factors of the studies. For any criterion which was not fulfilled according to the information outlined in the article, one point was deducted from a maximum of 20. Then the scores were calculated by scoring percentile. The qualitative scores were assessed by two independent investigators, and any disagreement was resolved by discussion.
